# Cross-linked decellularized porcine corneal graft for treating fungal keratitis

**DOI:** 10.1038/s41598-017-08207-3

**Published:** 2017-08-30

**Authors:** Yongliang Lin, Qinxiang Zheng, Shanshan Hua, Yongchun Meng, Wei Chen, Yifei Wang

**Affiliations:** 1 Biomedical Research and Development Centre, Jinan University, Guangzhou, 510632 China; 2 School of Ophthalmology and Optometry, Wenzhou Medical University, Wenzhou, 325027 China; 3 Ningbo First Hospital, Ningbo, 315010 China

## Abstract

This study aims to develop a cross-linked decellularized porcine corneal graft (cDPC) as a substitute for lamellar donor corneas and to evaluate the feasibility of using cDPC to treat fungal keratitis. The cDPC was prepared by decellularization, chemical crosslinking and γ-ray irradiation. Transparency, effectiveness of decellularization and biomechanical strength of cDPC were evaluated. The safety and efficacy of using cDPC to treat fungal keratitis were evaluated in the rabbit model. The transparency of cDPC was similar to that of a native porcine cornea (NPC), and no intact cells were observed in cDPC except for an insignificant amount of residual shrinking cellular nucleus. Compared to the NPC, the biomechanical strength of the cDPC was significantly increased. In the rabbit model of lamellar keratoplasty, the implanted cDPC reduced the incidence of corneal perforation, and also maintained transparency in majority. The results of this study suggest that the cDPC is capable of restoring the original transparency of cornea while effectively treating fungal keratitis. The cDPC is a highly promising ideal substitute for the donor human cornea.

## Introduction

Disease affecting the cornea are a major cause of blindness worldwide, second only to cataract in overall importance, ocular trauma and corneal ulceration are significant causes of corneal blindness that are often underreported, but may be responsible for 1.5–2.0 million new cases of monocular blindness every year^1^. Fungal keratitis as a cause of corneal ulceration is often neglected, especially in developing countries^2^. The standard first-line of treatment for fungal keratitis involves the local or systemic use of antifungal agents. However, many patients fail to respond to drug therapy and require keratoplasty.

Keratoplasty is performed by replacing the damaged or diseased cornea with a human cornea from a cadaver^3^. The first keratoplasty was performed in 1905^4^ and has been very successful with continued advancement of surgical techniques. Unfortunately, the number of donor corneas often are inadequate for patients needing keratoplasty. Demands for substitutes of donor corneas for use in transplantation have promoted the development of various materials intended for restoring corneal visual function^5^. In the development of corneal substitutes, properties of the cornea, such as transparency, biomechanical performance, biocompatibility, immunogenicity, and biotoxicity and inflammatory response should be taken into consideration^6^.

Recent progress has been made by using porcine decellularized corneal stroma, whose composition and micro-structure are similar to that in the human counterpart. Many studies have focused on the acellular corneal scaffold material. Wang *et al*. described the preparation of an acellular porcine corneal stroma (APCS) by decellularization with phospholipase A2 and demonstrated that APCS performed well as a scaffold material during rabbit lamellar keratoplasty^7^. In 2013, Kim *et al*. constructed a tissue-engineered cornea consisting of amniotic epithelial cells and an acellular porcine cornea. Their findings demonstrate that the tissue-engineered cornea has a great potential for the repair of severe corneal injury^8^.

The primary cause for graft rejection following keratoplasty with an allogeneic cornea is the residual corneal cellular components, which act as antigens presented by the major histocompatibility complex (MHC)^9, 10^. Therefore, it is important to eliminate the immunogenicity of cellular components by decellularization of the corneal graft. Conventional decellularization techniques include physical^11^, chemical, or enzymatic (phosphatase, collagenase^12^, etc.) processes, or any combination thereof. However, the decellularization process may reduce the biomechanical strength^13^ and transparency of APCS.

The development of a decellularized porcine cornea (DPC) would be ideal as it would maintain normal biomechanical properties and transparency by conforming to the existing extracellular matrix (ECM). Bearing this in mind, we prepared DPC using a chemical crosslinking procedure (1-ethyl-3-(3-dimethylaminopropyl) carbodiimide (EDC)/N-Hydroxysulfosuccinimide (NHS) crosslinking) to produce a cross-linked decellularized porcine cornea (cDPC) without the introduction of exogenous toxic substances. EDC/NHS crosslinked DPCs have excellent biomechanical and biocompatibility properties while maintaining transparency^14–16^.

Here, we demonstrated the development of a decellularized xenogeneic cornea as a substitute for human corneal donor grafts. In this study, cDPC were prepared by decellularization with a gradient osmotic pressure process, followed by EDC/NHS crosslinking and γ-ray irradiation. The cDPCs were tested as donors in the rabbit fungal keratitis model and evaluated for safety and efficacy.

## Results

### General morphology of cDPC

**Figure 1:**
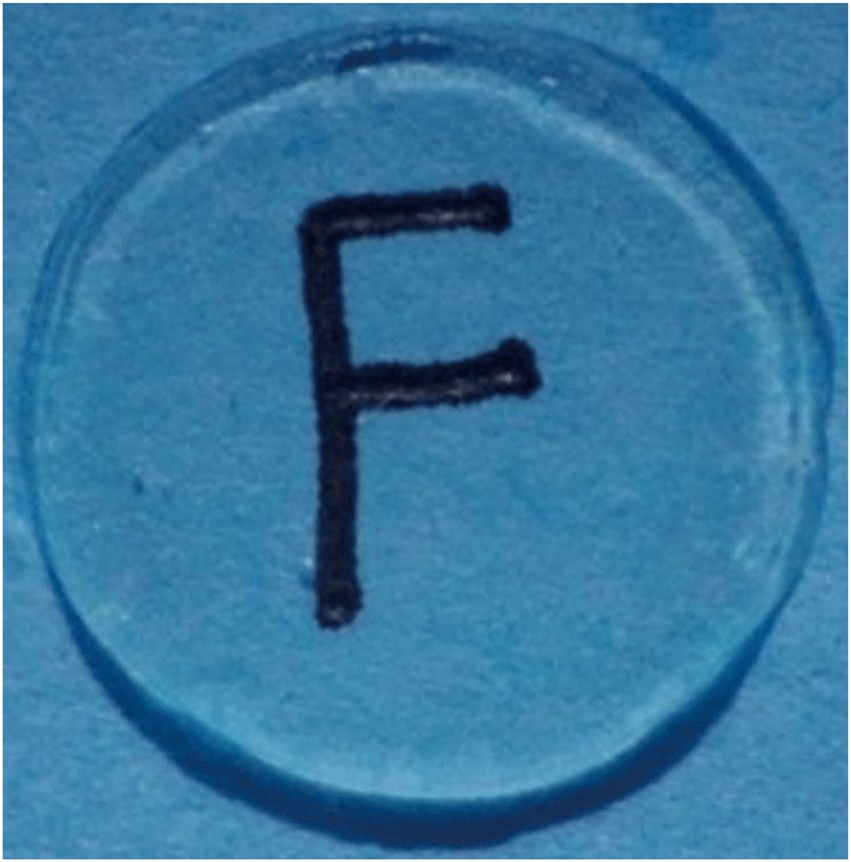
Appearance of the cDPC graft after preparing procedures including decellularization, crosslinking and γ-irradiation. The transparence of the cDPC is indicated by the letter “F” which can be obviously observed through the graft.

In order to examine the general appearance of the cDPC prior to transplantation, we examined cDPC by light microscopy. As seen in Fig. 1, in terms of general morphology, the cDPC graft remained transparent after the graft preparation procedure (i.e., decellularization, crosslinking and γ-ray irradiation).

### Light transmittance evaluation of transparency

**Figure 2:**
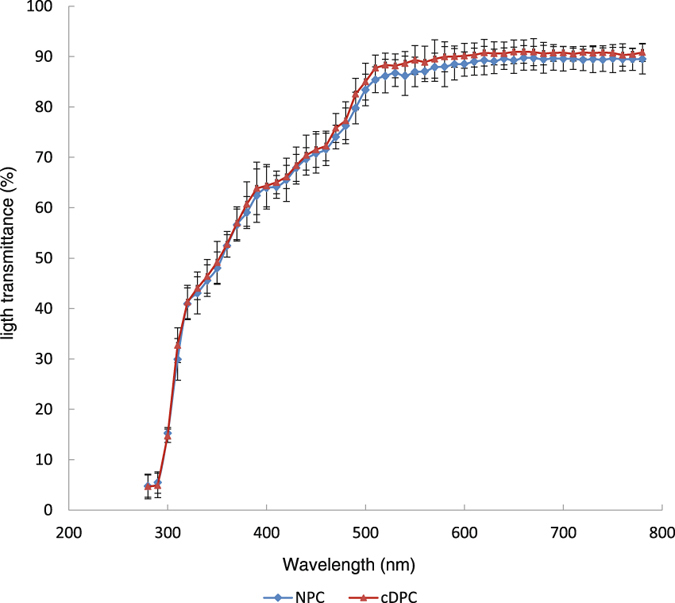
Light transmittance of the cDPC and NPC groups when wavelength ranges from 280 nm to 780 nm with a scanning interval of 10 nm.

To obtain a quantitative measure of light transmittance (LT), we measure LT over a range from 280 nm to 780 nm using an ultraviolet spectrophotometer. There was no significant difference between the NPC and cDPC groups at any wavelength between these two different wavelengths (n = 10, *p* > 0.05) (Fig. 2).

### H&E staining evaluation of structural integrity

**Figure 3:**
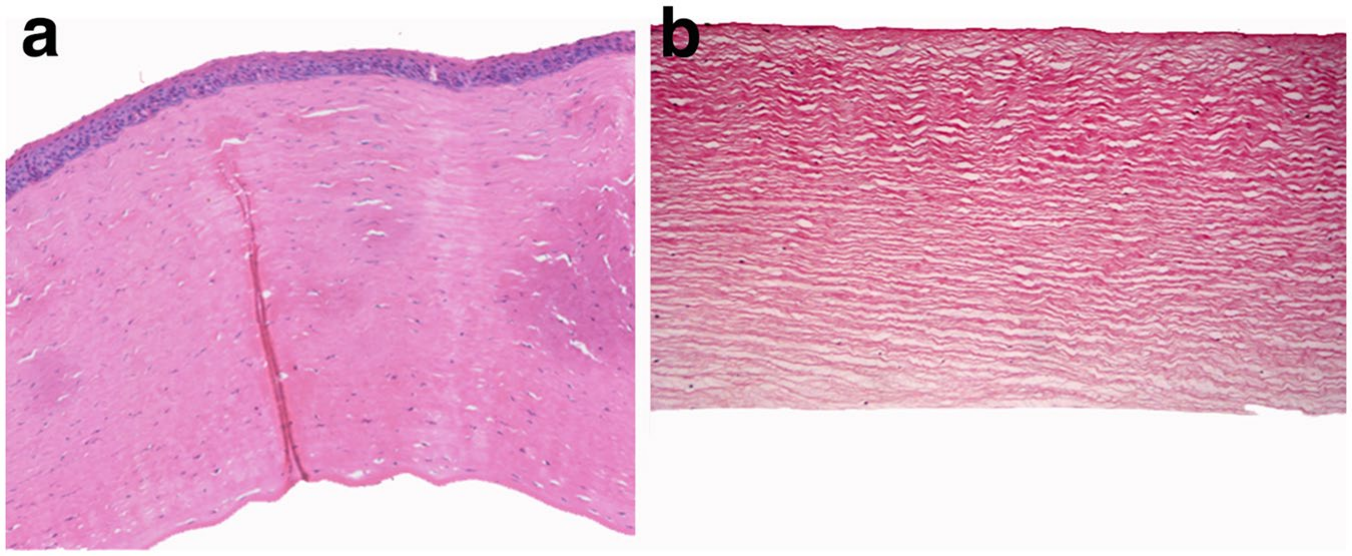
HE staining of NPC (**a**) and cDPC (**b**) (100× magnification).

Nuclear staining indicated that, under 100× magnification, corneal epithelial cells, stromal cells, and its endothelial cells were distinctly visible in the NPC group. The stromal collagen fibers appeared to have a normal arrangement, and an abundance of blue-dyed cells with intact structures were observed in the stroma. However in the cDPC group, no intact epithelial or endothelial cells could be observed except for an insignificant amount of residual shrinking cellular nucleus, while the stromal collagen fibers appear to have a normal orderly arrangement as observed in the NPC group (Fig. 3).

### Residual DNA Amounts following decellularization

In order to assess the effectiveness of decellularization, we measured the residual amount of DNA that remained on cDPC after processing, and compared it to that in the NPC. The DNA amount of the NPC group (469.97 ± 155.51 ng/mg, n = 6) was much higher than that in the cDPC group (82.16 ± 7.24 ng/mg, n = 6) (*p* < 0.05).

### Denaturation temperature

**Figure 4:**
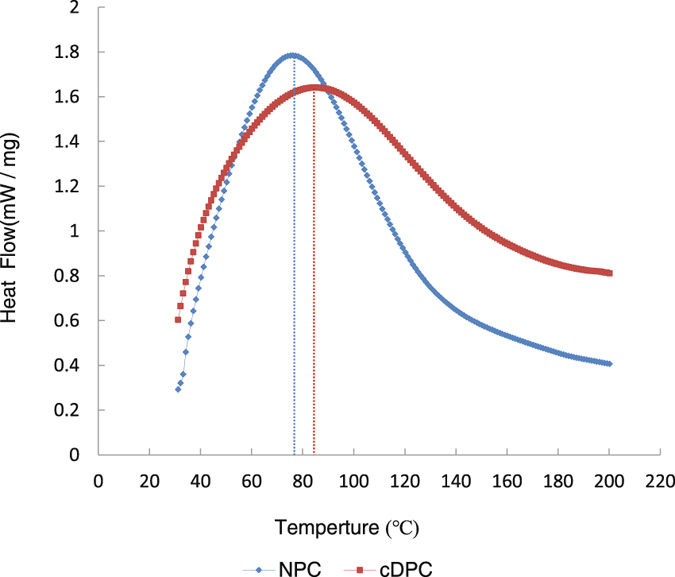
The denaturation temperature of the NPC (blue) and cDPC (red) grafts.

Compared to the NPC group (Td = 76 °C), the denaturation temperature (Td) of the cDPC group increased (Td = 84 °C) (Fig. 4).

### Determination of swelling rates

**Figure 5:**
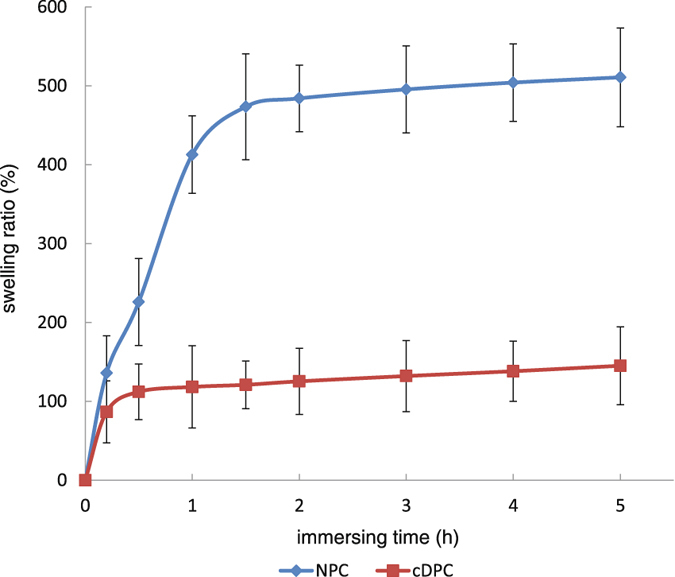
The swelling rates of the NPC and cDPC groups.

In order to determine the swelling rate of NPC and cDPC, we performed the assay as described in the Methods Section. Our results indicate that the highest swelling rates were observed during the first hour in both groups. Compared to the NPC group, the stabilized swelling rate of the cDPC group was significantly lower (*p* < 0.05) (Fig. 5).

### Tensile strength testing

**Figure 6:**
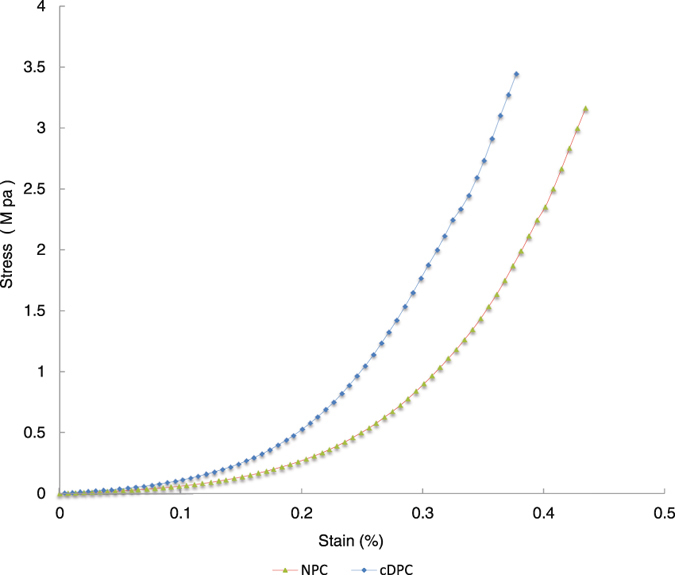
The typical stress-strain curve of NPC and cDPC groups.

The mean tensile strength of the cDPC group was 4.1750 ± 0.7438 Mpa (n = 10) which is significantly larger than 3.4775 ± 0.1584 Mpa (n = 10) in the NPC group. (*p* < 0.05). In contrast, the elastic modulus of the cDPC group was 5.75 ± 2.87 Mpa (n = 10), which was not significantly different from that of the NPC group (7.25 ± 2.75 Mpa, n = 10) (*p* > 0.05). The typical stress-strain curve of cDPC and NPC groups is shown in Fig. 6.

### Cytotoxicity

**Figure 7:**
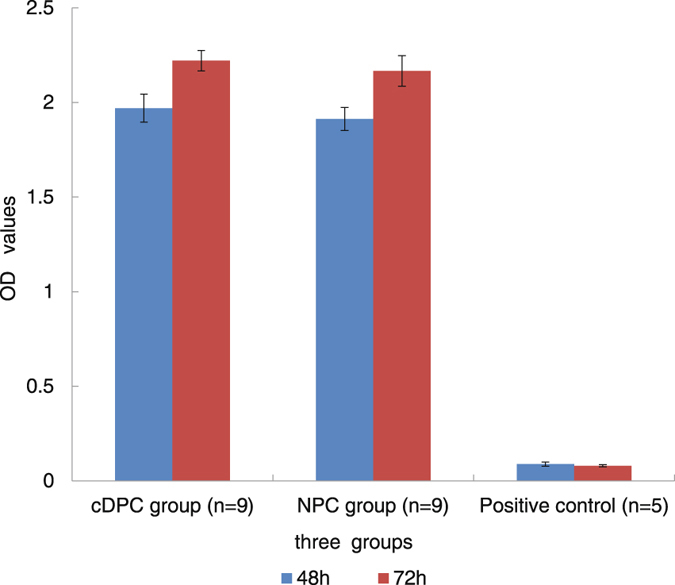
The results of cytotoxicity (measured by OD) of the cDPC group, and NPC group, and the positive control at 48 h and 72 h.

The optical density (OD) values after 48 h and 72 h incubation are shown in Fig. 7. There was no significant difference in the OD values between the cDPC group (n = 9) and the NPC group (n = 9) (*p* > 0.05). The MTT (3-(4, 5-dimethylthiazol-2-yl)-2, 5-diphenyl tetrazolium bromide) results showed that fibroblast L929 cells were able to proliferate in both cDPC and NPC groups, and there is no significant difference in cell proliferation between the two groups (p > 0.05) (Fig. 7).

### Establishment and validation of a rabbit fungal infection model

**Figure 8:**
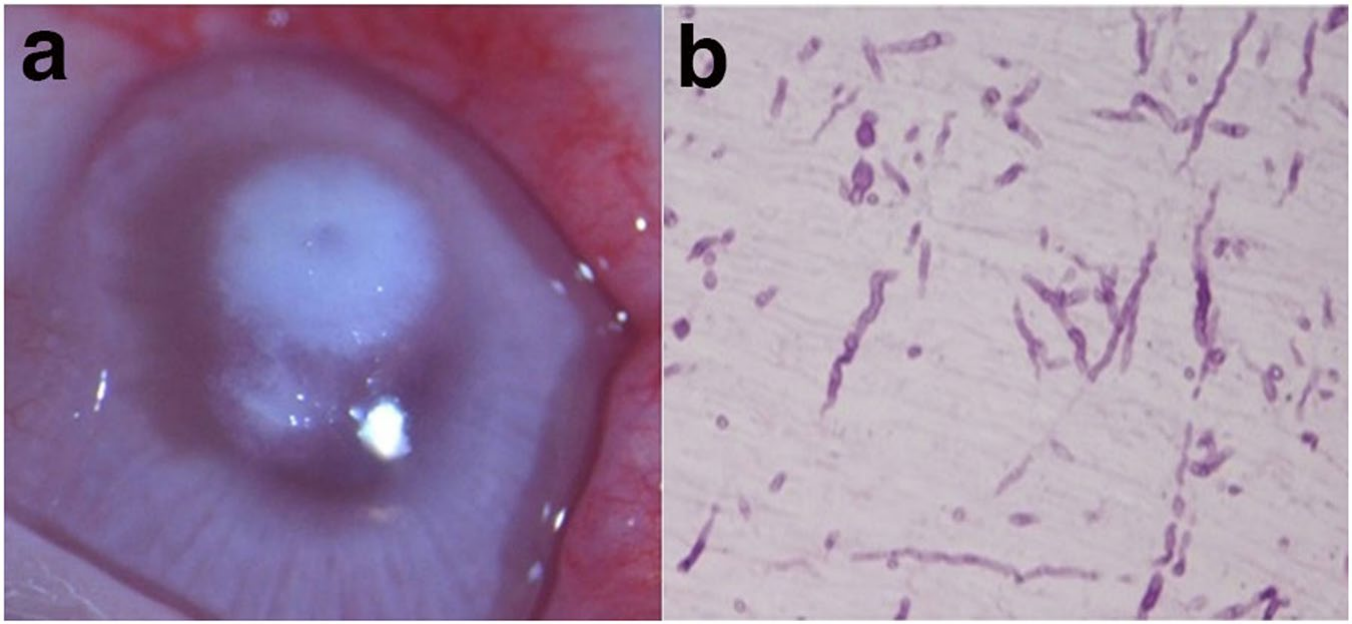
Images of the corneal fungal infection in rabbit model. (**a**) Anterior segment at the fifth day post-infection. (**b**) Histopathologic section image with PAS staining (which stains fungal hyphae).

By the fifth day post-injection with *Fusarium solani* into the rabbit corneal matrix, the focus of the injection spread to the peripheral areas with visible satellite focuses, pseudopodia, but no empyema was visible in the anterior chamber (Fig. 8a). PAS staining (which stains fungal hyphae) revealed abundant curved and relatively thick mycelium in the superficial stroma consistent with a fungal infection (Fig. 8b).

### Examination under slit lamp after fungal keratitis treatment

**Figure 9:**
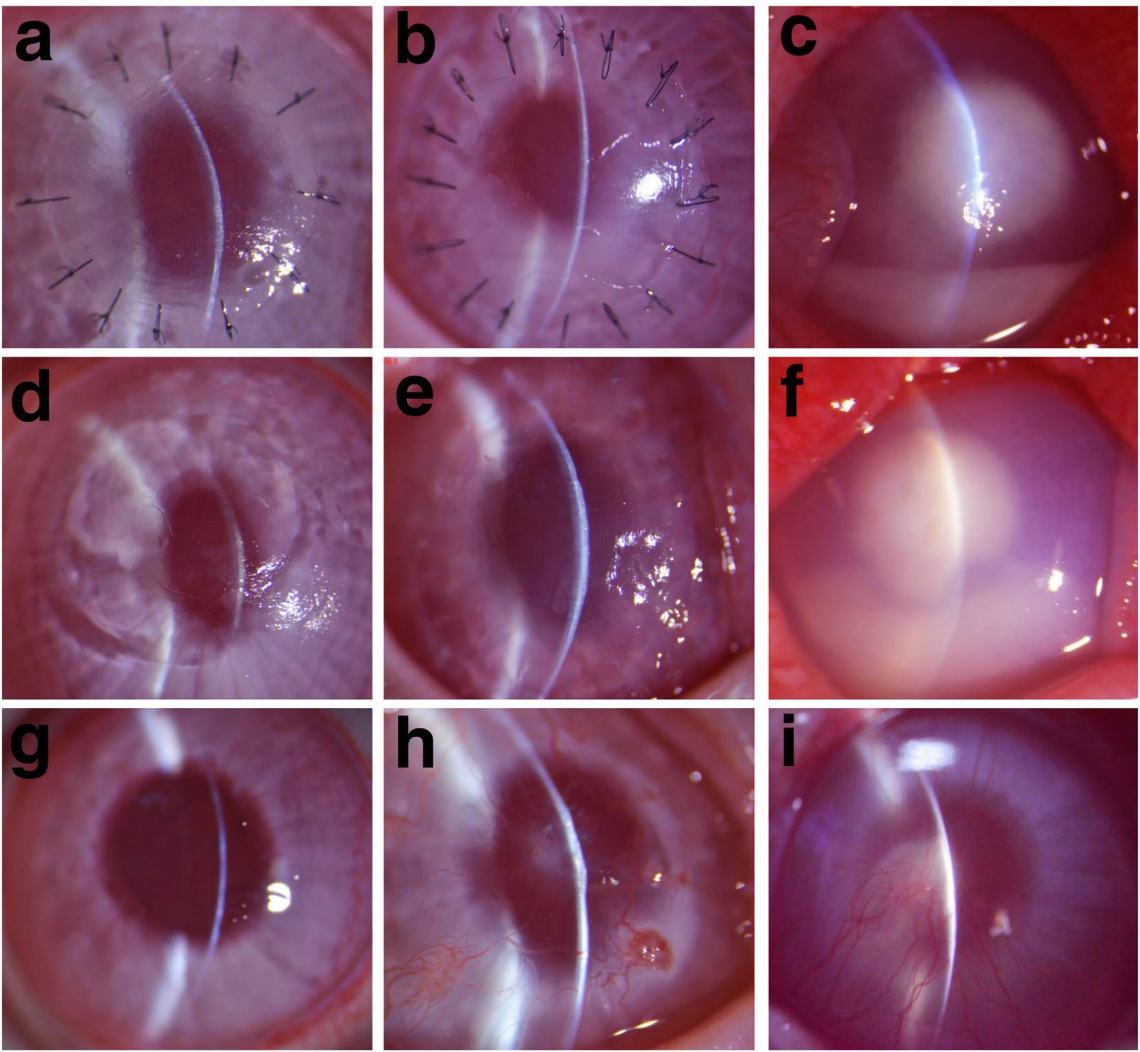
Examination under microscope post-surgery at one week (**a,b,c**), four weeks (**d,e,f**), 24 weeks (**g,h,i**) for fungal keratitis. (**a**) The cDPC presents mild corneal edema, neither cell in the anterior chamber nor sign of recurrent infection. (**b**) The NPC group has moderate edema with visible local inflammatory infiltration and no recurrent infection. (**c**)The Natamycin group presents a slight expansion of the infection focus, a significant increase in cellular infiltration, and a densified focus with a hypopyon that measured about 2 mm. (**d**) The cDPC graft shows neither visible corneal neovascularization nor inflammatory infiltration, while edema decreases and transparency improves. (**e**) The NPC graft shows increased corneal opacity, mild to moderate infiltration, and poor transparency. (**f**) The Natamycin group shows no significant expansion of focus, and has a large hypopyon (about 4 mm). (**g**) The peripheral cornea of the cDPC group shows little neovascular growth, neither inflammatory infiltration nor corneal edema, while the central cornea is almost transparent. (**h**) The NPC group shows dense white opacities in the central cornea with moderate neovascular growth and poor transparency. (**i**) The Natamycin group shows that the hypopyon decreases in size, but the central cornea remains cloudy with clear focus border while neovascular grows into the central cornea.

Lamellar keratoplasty was performed on both cDPC and NPC groups. As a control, another group was treated only with natamycin and was designated as the Natamycin group. Examinations under the slit lamp were performed after 1, 4 weeks, and 24 weeks post-surgery, respectively (Fig. 9).

### Healing status and complications after fungal keratitis treatment

Twenty-four weeks after fungal keratitis treatment was applied, the number of rabbits without fungal infection was 11 (91.7%) in the cDPC group (n = 12), 8 (66.7%) in the NPC group (n = 12), and 9 (75%) in the Natamycin group, respectively (n = 12). The remaining animals had corneal perforations.

### Histopathology after transplantation

**Figure 10:**
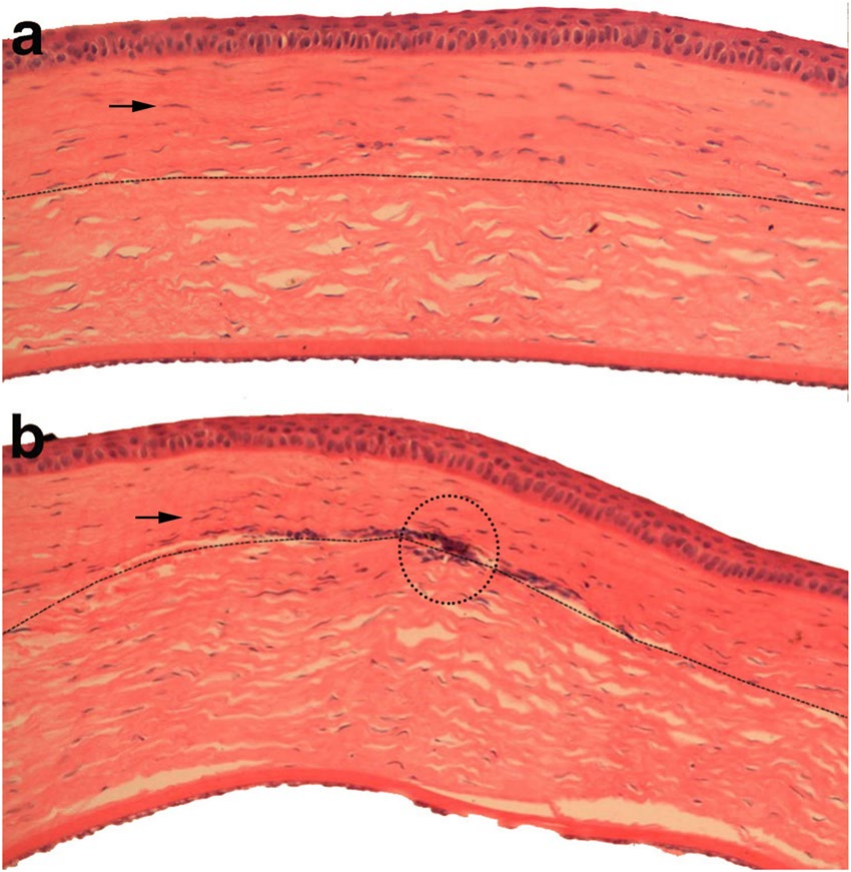
Corneal pathological section at twenty-four weeks after lamellar keratoplasty in Rabbit Model. (**a**) Histological analysis indicates growth of corneal epithelium in the cDPC graft with visible stromal cell growth (indicated by the arrow) into the superficial stroma of the corneal graft (above the dotted line). The image shows no infiltration of inflammatory cells at the graft periphery, and indicates overall good healing. (**b**) The NPC group shows abundant neovascular growth into the stroma (black circle).

Histomorphological observations of corneas at 24 weeks post-surgery are shown in Fig. 10.

### Long-term corneal transparency for fungal keratitis

The long-term results suggest that, in Natamycin group, corneal central opacity with significant neovascular growth into the central cornea can lead to severe vision loss. About 80% (7/9) had severe opacity through which no clear iris texture or pupil could be observed. The remaining animals had moderate opacity through which the pupil could be seen but the cornea was not clear.

The long-term results suggest that about 50% (4/8) of the eyes developed severe opacity in the transplanted NPC group and 37.5% (3/8) had moderate opacity. However, 72.3% (8/11) of the cDPC group had transparent grafts and only 18.2% (2/11) had mild opacity (Table 1).

**Table 1:** Corneal transparency for the cDPC, NPC and Natamycin groups. The total subject number of the cDPC, NPC and Natamycin group were 11, 8 and 9 respectively.

	cDPC group		NPC group		Natamycin group	
	Number	Percentage	Number	Percentage	Number	Percentage
Transparent	8	72.3%	0	0	0	0
Mild opacity	2	18.2%	1	12.5%	0	0
moderate opacity	1	9.1%	3	37.5%	2	22.2%
Severe opacity	0	0	4	50%	7	77.8%

## Discussion

Here, we describe the development of a novel method for preparing cDPC by decellularization with a gradient osmotic pressure procedure, combined with EDC/NHS crosslinking and γ-ray irradiation. Our results demonstrate that the combination of decellularization, crosslinking and γ-ray irradiation effectively reduces the immunogenicity while maintaining the transparency in both *in vitro* and *in vivo* experiments, and provides better biomechanical strength, thermal stability, and biocompatibility than other methods. The most important performance parameter of the corneal graft is transparency. Transparency of the corneal stroma depends on retention of the orderly lamellar arrangement of the collagen structure^17^. In this study, we utilized a more moderate and progressive gradient concentration of glycerol in the decellularization process, which protects the three-dimensional structure of the collagen fibers by preventing their disruption that accompanies fast and intense dehydration. In glycerol preservation solutions of high concentrations, the cornea is in an environment that prevents dehydration and the cornea effectively maintains its original shape, thickness and curvature. This precaution ensures that the collagen fibers maintain an arrangement close to a freshly harvested cornea. Likewise, we use slow and uniform crosslinking of the cornea in order to preserve the morphology of the cornea and the structure of collagen fibers. For reasons stated above, glycerol preservation is superior as it preserves the well-organized collagen fibers found in the fresh cornea and provides a transparent cDPC graft.

While 10kGy γ-ray irradiation disrupts collagen structure^18^, glycerol preservation effectively protects the glycosaminoglycan in the cornea and maintains the microstructure of the acellular matrix^19^ and thus maintains corneal transparency. Four weeks after surgery, epithelialization and restoration of endothelial cell function were observed in the cDPC group and was accompanied by a gradual decrease of the water content in the space between the collagen fibers allowing gradual recovery of corneal transparency^20^. Twenty-four weeks after surgery, the cDPC is completely transparent.

As cellular components are considered the main antigens involved in immune rejection, decellularization is the best way to overcome immunogenicity of donor tissue. The decellularization process often increases the space separating the collagen fibers and disrupts the original three-dimensional structure of the extracellular matrix (ECM)^21^. Additionally, residual detergent can be cytotoxic affecting cell regeneration. Thus, it is critical to balance complete decellularization and maintenance of ultrastructure of corneal collagen fiber. Such considerations are key to the successful preparation of donor corneal substitutes. To accomplish this goal, we developed a novel decellularization method: a gradient osmotic pressure formed by working solutions formulated with different glycerol concentrations.

The use of an osmotic pressure process with gradient glycerol concentrations effectively reduces the immunogenicity of the tissue as glycerol not only removes most of the cells, but also reduces the infiltration of inflammatory cells and reduces the incidence of rejection^22^. Glycerol disrupts cell membranes by destroying lipid components, eliminating or inactivating bacteria, fungi, and viruses^23^. At the same time, glycerol effectively protects the glycosaminoglycan in the cornea and maintains the structure of the extracellular matrix (ECM). Previous studies have reported that anhydrous glycerol preservation in a −78 °C freezer was a good method to preserve eligible tissues for deep anterior lamellar keratoplasty (DALK)^24^. Glycerol is useful as a hyperosmotic solution during osmotic treatment as it not only dissolves lipid membranes, removes membrane associated antigens, and soluble proteins and cells, but also changes the osmotic pressure and facilitates the removal of the ECM material. Our method of decellularization disrupts all the cell structure although a minimal amount of nuclear remains, which is less than the acquired amount (100 ng/mg) defined by the China Food and Drug Administration for the xenografts used in human tissues. Moreover, our compromise in complete cell removal leads to an optimal balance between the maintenance of ultrastructure of corneal collagen fibers and the maximal degree of decellularization.

The γ-ray irradiation is generally used as a method for sterilization of biological materials, but also effectively reduces the immunogenicity of the material^25^. γ-ray irradiation works by directly destroying DNA and causing indirect damage via reactive oxygen species (ROS) generation^26, 27^. Recent studies have reported that the γ-irradiated sterile decellularized cornea can be effectively applied to lamellar keratoplasty^28–30^. The combined use of polyethylene glycol and γ-ray irradiation makes the maximal decelluarization effect, because neither porcine endogenous retroviral DNA sequence nor alpha-1,3 galactose epitope was detected after treatment^31^. Histological examination confirmed that cells were extensively removed and the DNA residual amount significantly decreased in cDPC, while the ultrastructure of the collagen fibers remained nearly unchanged and similar to that of the NPC group. These results demonstrate that the process of decelluarization did not disrupt the corneal stroma. Among lamellar keratoplasty of cDPCs in rabbits, there was neither evidence of inflammatory cell infiltration, nor corneal vascularization.

Although decellularization of xenografts removes nearly all the cellular antigens and prevents immune rejection, the biomechanical strength and biostability of the material is often compromised. In order to improve the biomechanical strength and biostability of the decellularized porcine cornea, we employed a chemical process of crosslinking using EDC/NHS. In this crosslinking process, no new substances were introduced^32–34^, and the results of *in vitro* and *in vivo* cytotoxicity experiments have shown that the EDC/NHS cross-linked biosynthetic implants were not cytotoxic.

In order to verify that the crosslinking reaction occurred after EDC/NHS treatment, the thermal denaturation temperature of the collagen fibers was measured by DSC (Differential Scanning Calorimetry). A higher thermal denaturation temperature indicates a more stable material and more complete crosslinking^16^. Our results showed that the thermal stability of the cross-linked EDC/NHS sample was significantly higher than that of the un-crosslinked sample, which indicates that crosslinking effectively improved the physical properties of the cDPC.

Corneal swelling resulting from water imbibition affects its surface regularity, refractivity, surgical suture, cell migration, and tissue healing, while acellular tissue swells more easily than cellular tissue. However, our swelling experiments suggest that after crosslinking with EDC/NHS, the swelling rate of the cDPC was significantly lower than that in the NPC group. This suggests that crosslinking makes cDPC more resistant to edema than NPC.

The biomechanical properties of the cornea are directly related to the preservation and alignment of the matrix^35^. Many of the synthetic biomaterials previously reported as potential corneal substitutes failed to withstand interrupted sutures and stitch removal, and were not structurally strong enough. Previously reported acellular natural biomaterials also were not structurally sound after removal of cellular components^23^. The results of our study suggest that the cDPC underwent an increase in tensile strength that was significantly greater than the NPC group. However, we observed no significant difference in the elastic modulus between the cDPC and NPC groups. This difference may be caused by the crosslinking-induced connections formed between the collagen fibers. Such an effect, also explains the increased biomechanical strength of cDPC and the ability of the graft to withstand the surgical procedure.

Thus, our study suggest that EDC/NHS crosslinking is a crucially important step in the cDPC preparation process. However, excessive crosslinking could result in an increase in the rigidity and elastic modulus of the cDPC which may not comply with the clinical requirements. Therefore, the optimum EDC/NHS concentrations for crosslinking reactions should be further explored.

Xie *et al*. and others have established some animal models of fungal corneal infection^36^. We are the first to establish a rabbit model of fungal keratitis to evaluate the safety and efficacy of cDPCs in treating fungal keratitis. Our results demonstrate that the recurrent fungal infection rate in the cDPC group is significantly lower than that in the NPC group or the Natamycin group. Only one case of corneal perforation was observed in the cDPC group due to fungal infection recurrence. This resulted from primary disease recurrence after surgery of infectious keratitis, but was not a complication related to the keratoplasty. The NPC group had the lowest cure rate of fungal infections with four cases of perforation. The implantation of NPC without decelluarization led to early rejection due to intense local inflammation, eventually leading to corneal perforation. In the case of the Natamycin group, natamycin is a hydrophobic fat-soluble drug that has limited permeation in the cornea and has poor results for treating the fungal infection in superficial and deeper cornea. Eventually corneal fungal hyphae passed through the Descemet’s membrane into the anterior chamber to cause perforation.

Our study also demonstrates that, in addition to controlling fungal keratitis, the corneal transparency of cDPC group was obviously improved compared to the other two groups. Corneal transparency is crucial for a good prognosis after keratoplasty and has a direct effect on patient regaining or maintaining normal visual function. It is important to note that while the Natamycin group had a higher cure rate of fungal infections than NPC group, but corneal transparency was very poor. Long-term observations showed that all the corneas of the Natamycin group developed vascular scars during healing that led to moderate to severe corneal opacity. This often results in loss of visual function and induction of neovascularization that seriously damages the immune privilege of the cornea. Due to high antigenicity, the grafts of the NPC group were rejected and led to moderate to severe opacity. In the cDPC group, the post-surgery cornea remained transparent due to very low antigenicity. Crucial to long-term survival of the cDPC grafts, proliferation of rabbit corneal stroma cells and restoration of corneal transparency occurred after 24 weeks.

## Methods

### Cornea graft preparation

Fresh porcine eyeballs were obtained from local abattoirs (Guangzhou Animal and Husbandry Corporation, Guangzhou, China) and rapidly cooled. The ocular globe was washed three times with phosphate buffered solution (PBS) containing antibiotics (penicillin, 60 mg/L; streptomycin, 100 mg/L; Invitrogen, Carlsbad, CA, USA). Corneas were divided randomly into two groups: native porcine cornea (NPC) group and cDPC group. The NPC group consisted of fresh porcine corneas without further treatments. The cDPC group consisted of porcine corneas prepared by decelluarization, crosslinking and irradiation processes, as described below.

The prepared ocular globe was immersed in a gradient glycerol solutions with sequential concentrations of 95%, 70%, 50%, and 90%. The ocular globe was placed in each of these glycerol solutions for four hours at 4 °C with shaking, in order to ensure decellularization. The ocular globes were then washed with a preservation solution (500 g/L glycerol, 10 g/L hyaluronic acid, buffered salt solution).

Crosslinking was achieved using a glycerol buffered solution containing NHS and EDC (at a ratio of 1:2). The ocular globes were treated with this solution for two hours and washed with the preservation solution described above.

The anterior lamellar stroma was cut into a graft (250 μm in thickness and 10 mm in diameter from the eyeball) with a microkeratome (AMADEUS, Ziemer Corporation, Switzerland), Grafts were sealed in sterile plastic containers containing the preservative solution, irradiated with γ rays at 25 KGy, and stored at −20 °C or less until being used. All procedures were conducted under sterile conditions.

### Light transmittance assay

The cDPC and NPC grafts were immobilized on black corneal holders and placed in a spectrophotometer (U-4100, Shimadzu Ltd., Kyoto, Japan) for measuring transmittance. Transmittance was measured in the wavelength range from 280 nm to 780 nm at a scanning interval of 10 nm^7^.

### Differential scanning calorimetry

Differential scanning calorimetry (DSC Q2000, TA instruments Ltd., New Castle, USA) was used to obtain thermal properties of corneal grafts. Grafts of the cDPC and NPC groups were freeze-dried before determination. Then the samples of the cDPC and NPC grafts were heated at temperature ranging from room temperature to 200 °C (with an increasing rate of 10 °C /min) with a nitrogen flow rate of 50 mL/min, by setting an empty pan as a reference. Denaturation temperature (TD) was determined as the peak value of the corresponding endothermic phenomena.

### Swelling assay

The cDPC and NPC (n = 10) were dried at 37 °C in a vacuum oven for 24 h. The weight of each graft (W_d_) was measured with an electronic balance. The graft was immersed in 10 ml of physiologically isotonic PBS and the wet weight (Wt) was measured at 0.2 h, 0.5 h, 1 h, 1.5 h, 2 h, 3 h, 4 h and 5 h. The swelling rate (Q) was calculated by the following Eq. (1)^37^.

1 $$Q=\frac{{W}_{t}-{W}_{d}}{{W}_{d}}\times \mathrm{100} \% $$

### Histological staining

The graft was fixed in 4% paraformaldehyde overnight before embedding in paraffin. The 5 µm thick sections were stained with hematoxylin and eosin (H&E) and observed under a light microscope (Imager Z1, Zeiss, Oberkochen, Germany).

### DNA content

The cDPC and NPC grafts were dried and weighed, and then incubated in a proteinase K digestion solution (2 mg/mL, Takara, Otsu, Japan) for 12 h or longer at 56 °C. The digestion solution was centrifuged and the supernatant was collected. DNA was purified by using magnetic Beads Based DNA Extraction (Omega Bio-tek, Doraville, GA, USA). To quantify DNA, a Quant-iT^TM^ PicoGreen®ds-DNA assay kit (Life Technologies, Billerica, USA) was used according to the manufacturer instructions. The purified DNA was mixed with the PicoGreen reaction solution (Life Technologies, Billerica, USA) and the DNA content was quantitatively determined by using fluorescent microplate reader (LB942, Berthold Technologies, Wildbad, Germany) at an excitation wavelength of 485 nm and an emission wavelength of 535 nm.

### Tensile strength testing

The mechanical properties of the cDPC and NPC grafts were assessed via tensile testing (n = 10/group). To accomplish this purpose, grafts were cut into rectangular specimens with a gauge length of 9 mm and width of 2.5 mm, the thickness of samples was determined by a handheld thickness gauge (MZ2041, Jiangsu Mingzhu Testing Machinery Ltd., Jiangsu, China). The tensile strength was measured at a loading rate of 0.1mm/s by using an ElectroForce Test Instrument (ElectroForce 3200, Bose Corporation, USA) at the room temperature^38^. Force was applied until fracture occurred in the Test Instrument.

### Cytotoxicity of cDPCs

The cDPC(n = 9) and NPC(n = 9) grafts were extracted at 37 °C for 24 h with 10 ml extraction solution (DMEM supplemented with 10% fetal bovine serum, Gibco BRL, New York, USA) to obtain an extracted solution. The L929 fibroblast cells (4 × 10^4^/mL) were cultured in 23 wells (100 μl/well) of a cell culture plate and incubated at 37 °C with 5% CO_2_ for 24 h. The extracted solution (200 µl/well) was added in 18 wells, while DMSO was added to another 5 wells as a positive control. Cells were cultured, and the status of cell proliferation was quantitatively determined by MTT incorporation at 48 h and 72 h. The cell proliferation activity (OD value) was measured by a microplate reader (Bio-tekELX800, BioTek, Vermont, USA) at an absorbance value of 490 nm.

### Ethics statement

All animal experiments were carried out in accordance with the approved guidelines of the Wenzhou Medical University Institutional Animal Care and Use Committee. Forty Healthy adult New Zealand White Rabbits were obtained from Animal Center of Wenzhou Medical University (License No. SYXK 2015-0009). The experimental protocol was approved by the Wenzhou Medical University Animal Care and Use Committee (Permit Number: wydw 2016-0344).

### Establishment of a rabbit model of fungal keratitis

Healthy adult New Zealand White Rabbits of both sexes, were subjected to general anesthesia. The *Fusarium solani* (China General Microbiological Culture Collection Center, Beijing, China) at a concentration of 10^7^ CFU/ml was injected into the central corneal stroma of the subject rabbit by an insulin syringe. The injection depth was about 1/3 rd of the corneal stroma. On the fifth day post-injection, the severity of the corneal infection was observed under a slit lamp (Chongqing Kanghua Science & Technology Corporation, Chongqing, China). The extent and depth of the fungal infection within the cornea was determined by Periodic Acid-Schiff (PAS) staining and histopathologic examination.

### Surgery

The 36 rabbits infected with *Fusarium solani* were divided into three groups randomly (n = 12 per group). The first group (cDPC group) was treated with cDPC lamellar keratoplasty. The second group (NPC group) was treated with NPC lamellar keratoplasty. The third group (Natamycin group) did not receive keratoplasty but was treated with natamycin eye drops (Shenyang Sinqi Pharmaceutical Corporation, Shenyang, China) four times per day.

The rabbits in the cDPC group and the NPC group were anesthetized and sterilized. The eyelids were kept open with an eye speculum, and the upper and lower rectus muscles were pulled and fixed with a suture. In the central area of the rabbit cornea, a 7.5 mm trephine was used to prepare a 7.5 mm-diameter circular lamellar receipt bed. The diameter of this region was greater than the range of the infected ulcer focus and the depth of which was about 2/3 rd of the corneal thickness. After the infection focus was removed completely, a 7.75 mm-diameter circular graft with a thickness of about 250 μm was cut from each of the prepared cDPC and NPC groups (12 per group) by a corneal trephine, with the epithelial surface facing outward. 12–16 stitches of *in situ* interrupted suture were placed with the tightness incision of the suture perfectly aligned and the knot buried in the cornea. Ofloxacin ophthalmic ointment (North China Pharmaceutical Group Corporation, Shijiazhuang, China) was applied post-surgery. After surgery eyes were treated with eye drops for four weeks, tacrolimus solution (Senju Pharmaceutical Corporation, Osaka, Japan) and natamycin four times a day, while ofloxacin ophthalmic ointment twice a day.

### Routine examination and histological analysis

Transplanted grafts were examined under a slit lamp at the 1 week, 4 weeks and 24 weeks after surgery. At 24 weeks post-surgery, all rabbits were sacrificed by euthanasia and eyeballs were collected for histopathological analysis.

### Statistical analysis

Statistical analysis was performed using SPSS18.0 software. All data were expressed as Mean ± SD and analyzed for statistical differences using a one-way Analysis of Variance (ANOVA) test (*p* < 0.05 was considered a significant difference).

### Data availability

The datasets generated and analysed during the current study are available from Dr. Wang on reasonable request.

## Conclusion

In conclusion, the introduced novel method of preparing a decellularized corneal graft (cDPC) by decellularization, chemical crosslinking and γ irradiation produces the cDPC graft which has high transparency, good mechanical properties, long-term stability, low immunogenicity, and good biocompatibility. The study demonstrates that in a rabbit model of fungal keratitis, the cDPC can effectively control the infection while recovering the original transparency of the cornea. The cDPC may be an potential substitute for DALK in patients with fungal keratitis.
